# Stovaine as a Spinal and Local Anæsthetic

**Published:** 1906-01-27

**Authors:** 


### STOVAINE AS A SPINAL AND LOCAL ANÆSTHETIC.

Mr. G. L. Chiene described to the Edinburgh Medico-Chirurgical Society his experience in the use of stovaine as a spinal anæsthetic. This preparation was introduced by Fourneau in 1904 and presents the advantages over cocaine that a solution containing it may be boiled without decomposition, while it is much less toxic and only slightly inferior as a spinal anæsthetic. After referring to the results of others, Mr. Chiene stated that in all his cases he had employed the method recommended by Tuffier. The stovaine alone or with adrenalin was obtained in 10 per cent. solution in sterilised bulbs. The syringe and needle having been boiled, avoiding any contact with soda, the required quantity of solution was drawn up through the needle which was then removed from the syringe and mounted on a special metal handle devised by the writer which greatly simplified its introduction into the spinal canal. After the insertion of the needle as in lumbar puncture for diagnostic purposes, the handle was removed and the syringe replaced. The piston was then withdrawn so as to allow some of the cerebro-spinal fluid to mix with the solution in the syringe, the injection being then made, the needle withdrawn and the puncture sealed with collodion. The quantity injected varied from 2 centigrammes in a child to 8 to 10 in adults. The patient experienced a numbness in the legs and in about seven minutes there was complete anæsthesia of the perineum and lower limbs generally extending as high as the umbilicus. Mr. Chiene had performed a considerable number of operations by this method, including those for hernia, transplantation of tendons, and arthrectomy of the knee and ankle. In some of his earlier cases anæsthesia was incomplete or of too short duration, probably due to an insufficient dose, but in no case did any ill-effects result, though a few patients suffered from headache. The patients do not require to be propped up during operation as with cocaine, and it was observed that they did not present the alarming appearance often associated with the latter drug, and were generally able to enjoy a meal soon after the operation. As a local anæsthetic stovaine was inferior to cocaine though it had the advantage of greater safety. It was not a vaso-constrictor and acted better when combined with hemisine, and Mr. Chiene had not found this combination liable to produce gangrene as stated by some authors.

